# Wen-Xin Decoction ameliorates vascular endothelium dysfunction via the PI3K/AKT/eNOS pathway in experimental atherosclerosis in rats

**DOI:** 10.1186/s12906-016-1002-7

**Published:** 2016-01-23

**Authors:** Tongda Li, Dongmei Li, Hui Xu, Huamin Zhang, Danli Tang, Hongxin Cao

**Affiliations:** 1 The Gu-Lou Hospital of Traditional Chinese Medicine of Beijing, Beijing, China; 2 Graduate School of China Academy of Chinese Medical Sciences, #16 Nan-XiaoStreet, Dongcheng District, Beijing, 100700 China; 3 The Institute of Information on Traditional Chinese Medicine, China Academy of Chinese Medical Sciences, Beijing, China; 4 Experimental Research Center of China Academy of Chinese Medical Sciences, Beijing, China; 5 State Administration of Traditional Chinese Medicine of the People’s Republic of China, Beijing, China

**Keywords:** Wen-Xin Decoction, Vascular endothelium, Protective effects, Atherosclerosis, PI3K/AKT signaling pathway

## Abstract

**Background:**

Nitric oxide (NO) is the most powerful vasodilator that inhibits leukocyte adhesion, platelet aggregation, and vascular smooth muscle cell proliferation. However, excessive NO can cause lipid peroxidation and direct endothelial cell damage. Therefore, investigation of the role of NO in artherosclerosis development is important. Wen-Xin Decoction (WXD) has been shown to relieve myocardial ischemia reperfusion injury and prevent leukocyte adhesion and invasion; in addition, it can accelerate angiogenesis and prevent platelet activation and aggregation. In this study, we focused on the NO pathway to further clarify the protective effects of WXD on the vascular endothelium in rat models of artherosclerosis.

**Methods:**

Wistar rats were randomly divided into a normal group (*n* = 10) and a model group (*n* = 75). Rat models of atherosclerosis were generated by intraperitoneal vitamin D3 (3 months) injections and administration of a high-fat diet (3 months with vitamin D3 and 2 months alone). The model rats were randomly divided into five groups (*n* = 15 each): model (saline), atorvastatin (4.8 mg/kg/d atorvastatin), high-dose WXD (9 g/kg/d), medium-dose WXD (4.5 g/kg/d), and low-dose WXD (2.25 g/kg/d) groups. Each group received continuous drug or saline administration (suspended liquid gavage) for 30 days, following which all animals were sacrificed. The ultrastructure and histopathological changes of vascular endothelial cells and the expression of PI3K/AKT/eNOS and iNOS in the thoracic aorta tissue were analyzed.

**Results:**

WXD increased NO levels, modulated the NO/ET-1 ratio, and promoted repair of the injured vascular endothelium in a dose-dependent manner. At a high dose, WXD regulated the NO/ET-1 ratio as effectively as atorvastatin; furthermore, it increased NO levels within the physiological range to prevent endothelial damage caused by excessive NO expression. Real-time polymerase chain reaction and Western blot analysis showed that WXD significantly upregulated the mRNA and protein expressions of PI3K, AKT, and eNOS mRNA and significantly increased AKT and eNOS phosphorylation.

**Conclusions:**

Our results suggest that WXD protects and maintains the integrity of the vascular endothelium by activating the PI3K/AKT/eNOS pathway, decreasing iNOS expression, and promoting the release of physiological NO levels.

## Background

In 2011, the World Health Organization (WHO) reported that cardiovascular disease is the leading cause of death worldwide, with the number of deaths from coronary heart disease being as high as 730 million. Studies have shown that the increase in morbidity and mortality in low- and middle-income countries will increase the number of worldwide deaths from cardiovascular disease to 2330 million by 2030 [1]. Therefore, the prevention of cardiovascular disease is an important topic of research.

Atherosclerosis (AS) is the most common cause of cardiovascular disease, resulting in insufficient blood supply to the coronary arteries and subsequent myocardial ischemia and hypoxia, which are fundamental pathological processes of coronary heart disease (CHD). The underlying mechanisms are very complex. Studies have been trying to interpret this disease by investigating various factors, including “endothelial injury” [2], “lipid infiltration,” “inflammatory response” [3], and “thrombosis.” However, it is clear that disruption of the integrity of the vascular endothelium and its dysfunction are part of the initial factors causing AS [4–8]. Therefore, the protection of endothelial function is significant for the prevention and treatment of AS.

Vascular endothelium-dependent systolic and diastolic function is the gold standard for evaluating endothelial dysfunction [9]. It is crucial to maintain a balanced nitric oxide (NO)/ET-1 ratio [10]. Abnormalities in the ET and NO signaling pathways cause vascular endothelial dysfunction, endothelial necrosis, and disruption of endothelial integrity, all of which will lead to inflammatory infiltration, thrombosis, plaque formation, and other serious consequences. NO is a bifunctional endothelial vasodilator. It is the most powerful vasodilator that inhibits leukocyte adhesion, platelet aggregation, and vascular smooth muscle cell proliferation [11]. However, excessive NO can react with superoxide anions and generate strong oxidizing peroxynitrite anions (ONOO^−^), which can cause lipid peroxidation and direct damage to endothelial cells. Therefore, it is important to investigate the role of NO in the development of AS. Studies have shown that the therapeutic effects of statins and nitroglycerin on CHD are based on the regulation of NO levels.

In traditional Chinese medicine, the main pathomechanism of coronary artery AS and myocardial ischemia syndrome is Heart-Qi Deficiency (HQD), which is a leading cause of death and disability worldwide [12]. Wen-Xin Decoction (WXD) is considered an effective treatment modality that acts by warming yang supplementing heart and promoting blood circulation resolving phlegm (WSPR); this was first recorded in the “Synopsis of the Golden Chamber,” written and optimized by Professor Hongxin Cao (China Academy of Chinese Medical Sciences, CACMS). WXD is widely used for the amelioration of HQD and the clinical treatment of CHD [13]. As a famous herbal remedy, WXD can significantly relieve coronary artery spasm, decrease plaque formation, increase the myocardial oxygen supply, and improve the heart function. A recent metabolomics study showed that WXD exhibits potential pharmacological effects by restoring multiple disturbed pathways to the normal state [14]. Research also confirms that WXD relieves myocardial ischemia–reperfusion injury through the inhibition of NF-κB and downstream ICAM-1 and MCP-1 expression and the prevention of leukocyte adhesion and invasion. In addition, WXD can accelerate angiogenesis and prevent platelet activation and aggregation. In this study, we focused on the NO pathway to further clarify the protective effects of WXD on the vascular endothelium in rat models of AS.

## Methods

### Experimental animals

Eighty-five male Wistar rats (5 weeks old; weight, 200 ± 20 g) were provided by the Experimental Animal Center of the Chinese PLA Military Academy of Medical Sciences [License No.: SCXK-(Army) 2007-004]. The rats were housed in the experimental animal center at the Institute of Basic Theory, CACMS. There were five rats per cage, with a total of 17 cages. All rats were provided free access to water and were maintained on a 12-h light/dark cycle, with a temperature of 20 ± 2 °C and humidity of 50 ± 5 %. The animals were cared for in accordance with the laboratory animal care protocols of the Institute of Basic Theory of Chinese Medicine, CACMS. All animal studies conducted here were approved by the medical ethics committee of the Institute of Basic Theory of Chinese Medicine, CACMS.

### Experimental reagents

The component herbs of WXD included the following: *Ginseng Radix et Rhizoma* (10 g), *Cinnamomi Ramulus* (10 g), *Allii Macrostemonis Bulbus* (15 g), *Trichosanthis Fructus* (15 g), *Pinelliae Rhizoma Praeparatum Cum Alumine* (10 g), *Paeoniae Radix Rubra* (15 g), *Ophiopogonis Radix* (15 g), and *Coptidis Rhizoma* (10 g). These were purchased from Beijing Tong-Ren-Tang Pharmacy and were certified as authentic by the Institute of Chinese Meteria Medica, CACMS. They also fulfilled the standard requirements of the 2010 version of the Chinese Pharmacopoeia. The traditional decocting method is described as follows. First, 1000 ml water is added for impregnation, the extract is heated for 30 min, and the liquid is leached. Then, 500 ml water is added to the residue, the extract is heated for 30 min, and the liquid is leached. The two portions of leached liquid are merged for liquid static sedimentation, filtration, and concentration. Finally, the derived product is packed for use. For controlled administration, we prepared WXD as a dry extract according to the guidelines of the School of Chinese Materia Medica, Beijing University of Chinese Medicine. To prepare the dry extract, the herbs were immersed, boiled, and filtered. The filtrates were combined and concentrated in a constant volume, steamed in a water bath until nearly dry, placed in an oven at 105 °C for 3–4 h, and cooled in a dryer for 0.5 h. concentrate leaching into the powder preparation. The extract concentration was 30.58 %, which was equivalent to 3.27 g of the original medicines per 1 g of dry extract. We used a certain amount of distilled water to dissolve the dry extract. The dry extract was prepared in strict compliance with the Chinese Pharmacopoeia ((see Appendix IO) and CGEP (GMP herbal extracts) in order to ensure the quality of medicines.

The other commercial products used in this study were as follows: atorvastatin calcium (Pfizer, Lipitor; code number approved by SFDA: J20070061), vitamin D3 (Shanghai General Pharmaceutical Co., Ltd.; code number approved by SFDA: H31021404; dose: 600,000 IU, 1 ml), AngII-ELISA kit (BG; E02A0204), ET-1ELISA kit (BG; E02E0040), NO-ELISA kit (BG; E02N0041), real-time quantitative polymerase chain reaction (real-time PCR) kit (SYBR Green PCR Mixture, CWbio. Co., Ltd., CW0957), rabbit anti-PI3K P85 (4292S; CST; dilution ratio 1:1000), rabbit anti-p-eNOS (9570; CST; dilution ratio 1:1000), rabbit anti-eNOS (ab11627; Abcam; dilution ratio1:300), rabbit anti-P-AKT (ab38449; Abcam; dilution ratio 1:500), rabbit anti-AKT (ab8805; Abcam; dilution ratio 1:500), rabbit anti-iNOS (ab15323; Abcam; dilution ratio 1:250), and a high-fat diet (4 % cholesterol, 10 % lard, 5 % sucrose, 81 % diet).

### Preparation of animal models

After 1 week of acclimatization, the Wistar rats were randomly divided into a normal group (*n* = 10) and a model group (*n* = 75). The total time taken for model preparation was 5 months. During the first 3 months, the rats received a high-fat diet combined with intraperitoneal injections of vitamin D3 (150,000 U/kg, once a month). For the next 2 months, they received the high-fat diet alone.

According to the AS index (AI), the 75 model rats were randomly divided into five groups of 15 each: model, atorvastatin, high-dose WXD, medium-dose WXD, and low-dose WXD groups. Each group received continuous drug (suspended liquid gavage) or saline administration for 30 days as follows: normal and model groups, saline (10 ml/kg/d); atorvastatin group, atorvastatin (4.8 mg/kg/d, equivalent to five times the adult human dose); WXD high-dose group, 9 g/kg/d WXD (equivalent to two times the human dose); WXD medium-dose group, 4.5 g/kg/d (equivalent to one time the human dose); and WXD low-dose group, 2.25 g/kg/d (equivalent to half the human dose). All animals were sacrificed after 30 days of drug or saline administration, with no food intake before sacrifice.

### Tissue preparation

On day 180, rats were anesthetized by intraperitoneal injection of 5 % urethane (1000 mg/kg), following which blood samples were collected from the abdominal aorta and the full-length aorta (from the aortic arch to the iliac artery bifurcation) was harvested. Blood samples from the abdominal aorta were drawn (10 ml), and the serum was collected and stored at −20 °C. Serum levels of cholesterol (CHO), triglyceride (TG), low-density lipoprotein cholesterol (LDL-C), and high-density lipoprotein cholesterol (HDL-C) were measured using an Automatic Biochemistry Analyzer (HICATHI) according to the manufacturer’s instructions. After removal of the epicardial adipose tissue, the aortic arch and 1 cm of the lower end of the thoracic aorta were harvested and placed in liquid nitrogen for real-time PCR and Western blot analysis. The coronary artery (1 mm^3^) was fixed in glutaraldehyde for observation under a transmission electron microscope. Part of rat aorta was examined by hematoxylin and eosin staining for histopathological changes.

### Real-time PCR

Total RNA was extracted by an ultrapure RNA extraction kit. In total, 8-μl RNA samples were used for 1 % agarose gel electrophoresis. Reverse transcription was performed using the HiFi-MMLV first strand cDNA synthesis kit (CWbio. Co., Ltd.). RNA templates (2 μl) were added to a 20-μl reaction system to achieve a final concentration of 10 ng. Real-time PCR was performed using the Bioer line-gene quantitative PCR device, and the products were amplified using the SYBR PCR Mixture according to the manufacturer’s instructions. The primers for AKT, eNOS, PI3K, actin, and iNOS were as follows: AKT, 5-AGC ATG GAG TGT GTG GAC AG-3 (forward) and 5-GAT GAT CCA TGC GGG GCT T-3 (reverse); eNOS, 5-AAG GCA AAC CAC CCT CTC TG-3 (forward) and 5-TTA GGC TGA CTC CCT CCC AG-3 (reverse); PI3K, 5-GCT CTT TCC CCA GCT GAA CT-3 (forward) and 5-ACA CAG TGT CGC TGG TTT GA-3 (reverse); actin, 5-CCC ATC TAT GAG GGT TAC GC-3 (forward) and 5-TTT AAT GTC ACG CAC GAT TTC-3 (reverse); and iNOS, 5-ACACAG TGT CGC TGG TTT GA-3 (forward) and 5-AAC TCT GCT GTT CTC CGT GG-3 (reverse). The reaction system was as follows: SYBR PCR Mixture (2×), 10 μl; forward primer (10 uM), 1.5 μl; reverse primer (10 uM), 1.5 μl; and template, 3 μl. All reactions were performed in triplicate. Gene expression was assessed using the 2^−△△CT^ method as follows: △△CT = △CT (drug treatment group) − △CT (normal group) and △CT = CT (target gene) − CT (reference gene).

### Western blot analysis

Total protein was isolated using ice-cold RIPA and protease inhibitor cocktail (Roche); phosphorylated protein extraction required phosphatase inhibitors. In total, 50 mg of tissues were homogenized using 500 μl of RIPA lysate buffer, incubated on ice for 20 min, and centrifuged at 13,000 rpm (4 °C) for 20 min. The supernatant was collected and stored at −80 °C. The protein concentration was determined using the BCA Protein Assay Kit according to the manufacturer’s instructions. Protein samples (30 μg) were added for 12 % polyacrylamide gel electrophoresis and transferred to a PVDF membrane. The membrane was blocked with 5 % skimmed milk at room temperature for 2 h, incubated with the primary antibody for 1 h at room temperature, and maintained at 4 °C overnight. The following day, the membrane was washed with TBST (six times, 5 min each) and incubated with the secondary antibody at room temperature for 1 h. After washing with TBST (six times, 5 min each), the membrane was developed in a darkroom. The gray scale of the protein bands was analyzed using ImageJ software.

### Statistical analysis

All data are expressed as means ± standard deviations (means ± SDs). Statistically significant differences between groups were determined using one-way ANOVA and Dunnett’s t-tests. The results of real-time PCR and Western blot analysis were analyzed using independent samples two-tailed t-tests. All data were analyzed using SPSS 16.0 software. A *p*-value of <0.05 was considered statistically significant.

## Results

### General condition of animals

Before model preparation, all animals were alert, responsive, and healthy. After model preparation, the rats in the normal group exhibited smooth fur, were supple and active, and ate well. Compared with the normal group, the rats in the model group were significantly underweight and less active and exhibited dull fur, significantly lesser food intake, teeth deformation, and nose bleeding. Compared with the model group, the rats in the WXD-treated groups ate well, were very active, and exhibited significant weight gain (Table 1) and smooth fur. Four rats in the model group and one in the WXD-treated group died during the experiment. Autopsy revealed that two of the four rats from the model group and the rat from the WXD-treated group died because of excessive anesthesia administered during blood sample collection from the eye vein on day 120. One rat in the model group was diagnosed with heart failure according to the presence of a pale heart, swollen and bleeding lungs, extremely low weight, and mouth bleeding, while inappropriate gavage was identified as the cause of instant death in the remaining rat.

**Table 1:** Changes in the body weight of rats in each group (mean ± SD, gram)

Group	*N*	T_0_	T_1_	T_2_
Normal	10	206.5 ± 8.4	571.3 ± 60.9	582.8 ± 48.2
Model	10	201.1 ± 11.0	500.1 ± 19.2*	435.6 ± 59.3*
L-WXD	10	208.4 ± 15.1	505.6 ± 39.1^#^	541.8 ± 34.3^#^
M-WXD	10	207.2 ± 7.8	510.5 ± 33.3^#^	568.4 ± 39.8^#^
H-WXD	10	206.6 ± 24.2	508.4 ± 31.8^#^	630.0 ± 62.4^#^
Atorvastatin	10	206.3 ± 9.1	500 ± 71.2^#^	507.1 ± 66.3^#^

Note: T_0_, −1 day; T_1_, 150 days; T_2_, 180 days

*p < 0.05, versus the normal group

^#^ *p* < 0.05, versus the model group

### Effects of WXD treatment on the endothelial cell ultrastructure

**Fig. 1:**
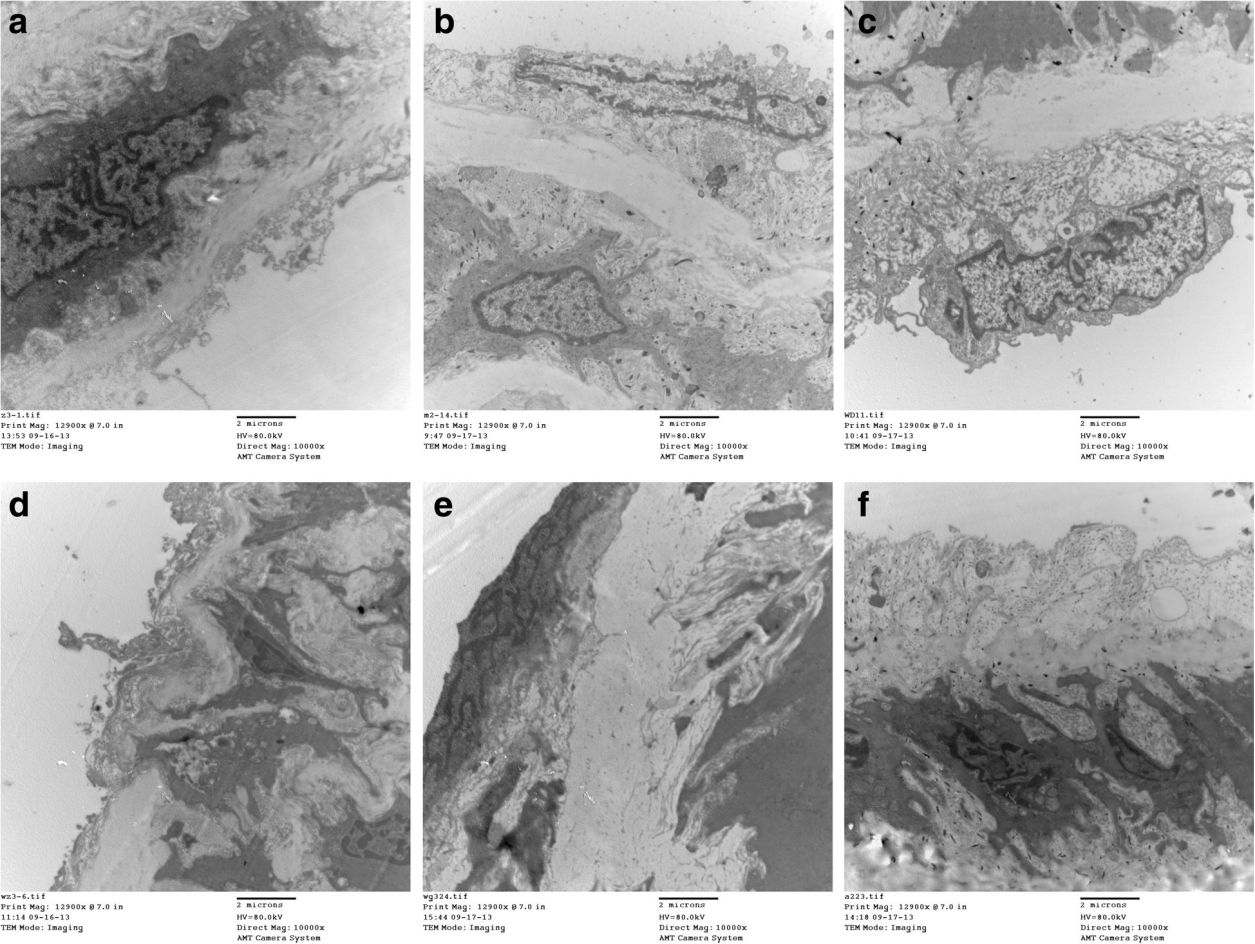
Changes in the structure and ultrastructure of the aortic arch as observed by transmission electron microscopy (magnification, ×10,000) after 30 days of treatment with Wen-Xin Decoction (WXD). **a** normal group; **b** model group; **c** low-dose WXD group; **d** medium-dose WXD group; **e** high-dose WXD group; **f** atorvastatin group

Transmission electron microscopy revealed significant ultrastructural changes in the endothelial cells, intimal and medial elastic membranes, and smooth muscle cells in the aorta from the model group. These included swelling and protrusion of the endothelial cells, elastic membrane loss, and branched smooth muscle cells. After WXD treatments, there was no significant protrusion and/or thickening of the thoracic aorta membrane, with no significant disruption in the integrity of the endothelial cells, elastic membranes, and smooth muscle cells. The high-dose WXD group exhibited the best therapeutic effects. In the atorvastatin group, the endothelial cells were swollen and dislodged, accompanied by loose connective tissue and disruption of the integrity of the smooth muscle cell layer. The overall therapeutic effects were better in the WXD treatment groups than in the atorvastatin group (Fig. 1).

### Effects of WXD treatment on histopathological changes in the thoracic aorta

**Fig. 2:**
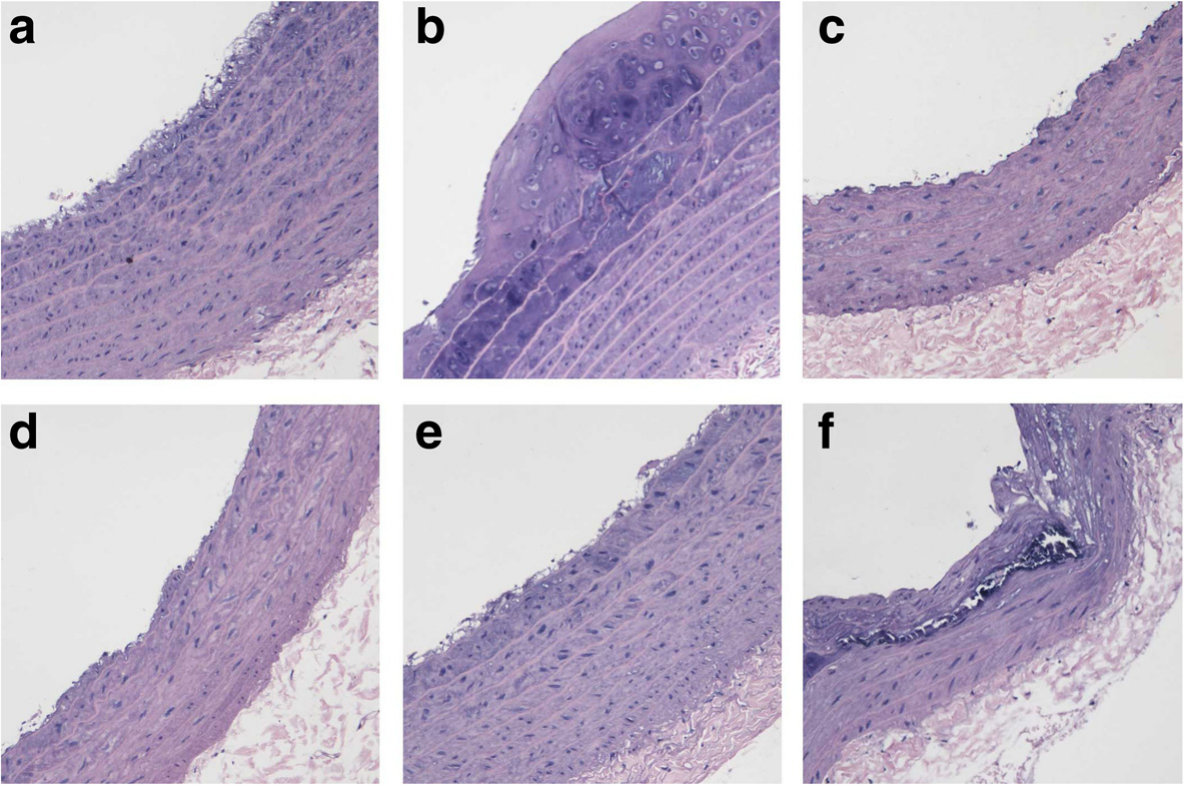
Effects of Wen-Xin Decoction (WXD) on histopathological changes in the aortic arch after 30 days of treatment (magnification, ×200). **a** normal group; **b** model group; **c** low-dose WXD group; **d** medium-dose WXD group; **e** high-dose WXD group; **f** atorvastatin group

Histopathological changes in the rat aorta were examined by hematoxylin and eosin staining (Fig. 2). Compared with the model group, the WXD and atorvastatin groups showed a visible decrease in pathological changes; the vessel walls were slightly rougher and thicker, and fewer atherosclerotic plaques were observed.

### Effects of WXD treatment on serum lipid levels

Compared with those in the model group, CHO, TG, and LDL-C levels were lower in the medium- and high-dose WXD groups (*p* < 0.05), while there was no significant difference between high-dose WXD group and atorvastatin group (*p* > 0.05). HDL-C levels (*p* < 0.05) were increased in the medium- and high-dose WXD groups (Table 2).

**Table 2:** Effects of Wen-Xin Decoction (WXD) treatment on serum lipid levels (mmol/L)

Group	*N*	CHO	LDL-C	TG	HDL-C
Normal	10	1.836 ± 0.266	0.314 ± 0.039	1.053 ± 0.142	1.258 ± 0.158
Model	10	3.861 ± 0.841***	1.436 ± 0.534***	2.706 ± 1.023***	0.884 ± 0.121***
L-WXD	10	3.530 ± 0.246^△△△^	0.935 ± 0.108^###△△^	2.208 ± 0.518^△△^	0.952 ± 0.117^△△△^
M-WXD	10	2.687 ± 0.456^#^	0.754 ± 0.195^###^	2.163 ± 0.316^△△^	1.094 ± 0.161^##△△△^
H-WXD	10	2.699 ± 0.488^#^	0.629 ± 0.131^###^	1.553 ± 0.236^###^	1.284 ± 0.255^###^
Atorvastatin	10	2.462 ± 0.198^##^	0.595 ± 0.127^###^	1.430 ± 0.247^###^	1.496 ± 0.144^###^

Note: values are expressed as mean ± SD

*p < 0.05,**p < 0.01, ***p < 0.001, versus the normal group

^#^ *p* < 0.05,^##^ *p* < 0.01, ^###^ *p* < 0.001, versus the model group

^△△^ *p* < 0.01, ^△△△^ *p* < 0.001, versus the atorvastatin group

### Effects of WXD treatment on serum NO levels and the NO/ET-1 ratio

**Fig. 3:**
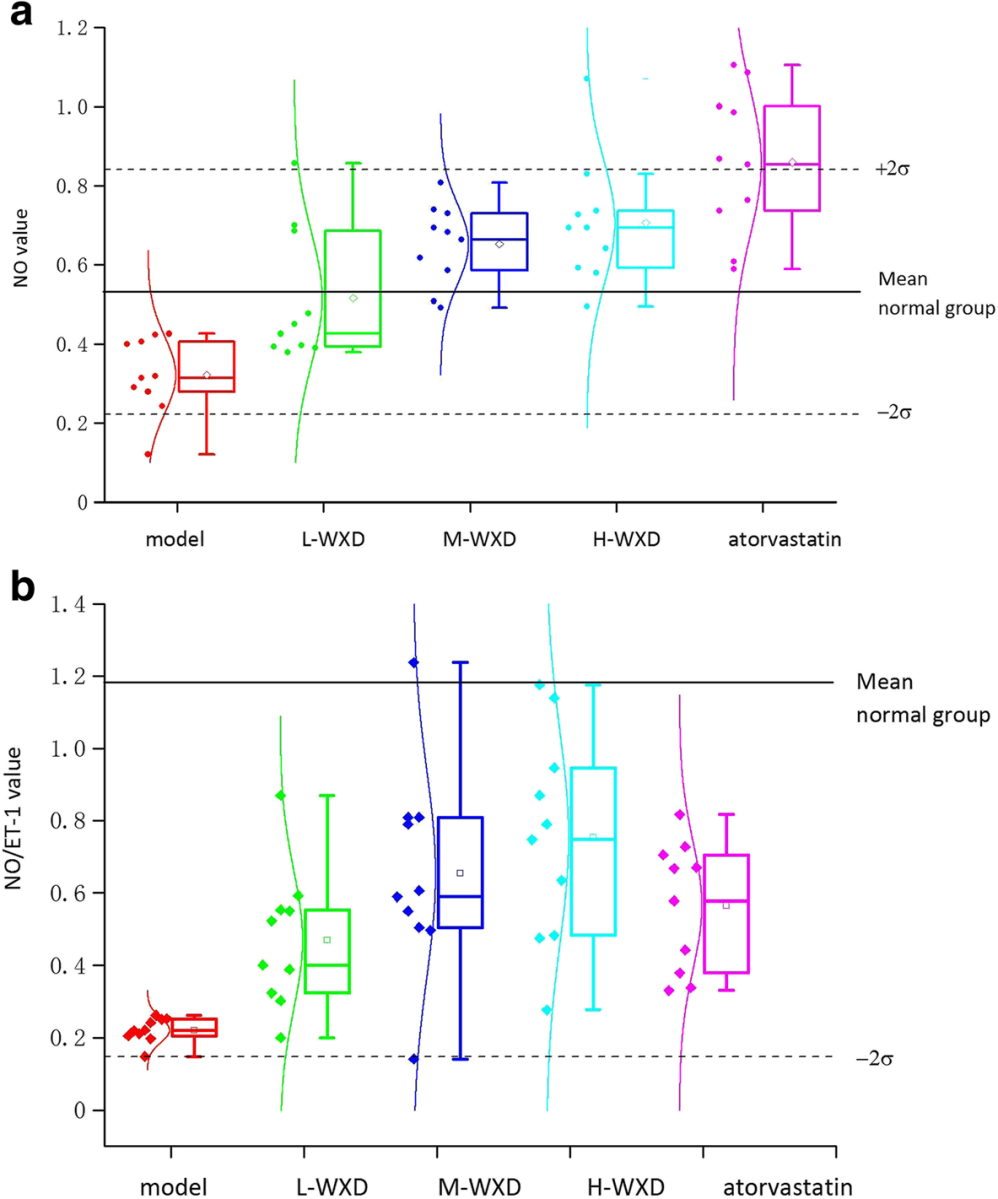
**a** Effects of Wen-Xin Decoction (WXD) on serum nitric oxide (NO) levels. **b** Effects of WXD on the serum NO/ET-1 ratio (*n* = 10)

Compared with those in the normal group, serum NO levels in the model group were significantly decreased, with the data distributed mostly within the [Mean − 2σ, Mean] range (*p* < 0.001; Fig. 3a). Compared with those in the model group, serum NO levels in the WXD treatment groups were significantly increased in a dose-dependent manner (*p* < 0.001, Fig. 3a), with data for the medium- and low-dose WXD groups distributed mostly within the [Mean, Mean + 2σ] range, indicating that WXD treatment significantly increased NO levels within the physiological range to protect the blood vessels (Fig. 3a). The atorvastatin group also exhibited a significant increase in NO levels compared with the model group (*p* < 0.001). However, most of the data exceeded the [Mean, Mean + 2σ] range; excessive expression of NO may damage the endothelium.

Compared with that in the normal group, the serum NO/ET-1 ratio was significantly decreased in the model group, indicating the dysfunction of diastolic vasoconstriction. Compared with that in the model group, this ratio was balanced in the WXD and atorvastatin groups (*p* < 0.001); the high-dose WXD group exhibited slightly greater effects compared with the atorvastatin group (*p* > 0.05; Fig. 3b). Although low-dose WXD treatment increased the NO/ET-1 ratio, there was no significant difference from the ratio in the model group (*p* > 0.05), indicating that the effects of WXD treatment on the NO/ET-1 ratio was dose dependent.

### Effects of WXD treatment on the PI3K/AKT/eNOS pathway

**Fig. 4:**
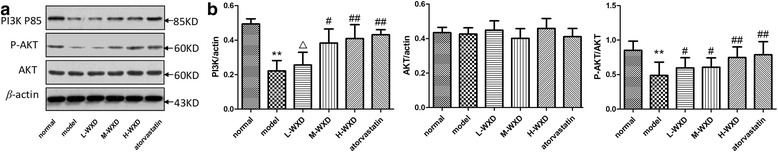
Protein expression of PI3K, AKT, p-AKT, and β-actin in the aorta after 30 days of Wen-Xin Decoction (WXD) treatment. Data are obtained from six rats. *p < 0.05, **p < 0.01 compared with the normal group. ^#^ *p* < 0.05, ^##^ *p* < 0.01 compared with the model group, ^△^ *p* < 0.05 compared with the atorvastatin group

**Fig. 5:**
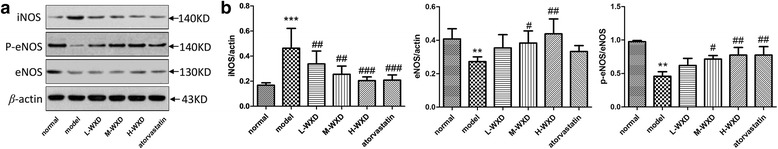
Protein expression of eNOS, p-eNOS, iNOS, and β-actin in the aorta after 30 days of Wen-Xin Decoction (WXD) treatment. Data are obtained from six rats. *p < 0.05, **p < 0.01 compared with the normal group. ^#^ *p* < 0.05, ^##^ *p* < 0.01 compared with the model group

High-dose WXD treatment significantly increased the protein expression of PI3K and eNOS and significantly enhanced the phosphorylation of AKT and eNOS, indicating an upregulation of the PI3K/AKT/eNOS pathway. Low-dose WXD treatment only increased AKT phosphorylation and had little impact on the total protein expression of PI3K, AKT, and eNOS. Medium-dose WXD treatment increased protein expression of PI3K and eNOS and phosphorylation of AKT and eNOS, although it had little impact on AKT expression. Atorvastatin treatment significantly increased PI3K protein expression and enhanced AKT and eNOS the phosphorylation. iNOS protein expression was downregulated in all treatment groups; however, the high-dose WXD and atorvastatin groups exhibited the best therapeutic effects. This indicated that the effects of WXD were dose dependent. The images of protein expressions of PI3K, AKT , p-AKT, eNOS, p-eNOS and iNOS are in Figs. 4a and 5a, and the results are presented in column diagram form in Figs. 4b and 5b.

### Effects of WXD on the gene expression of PI3K, AKT, eNOS and iNOS

**Fig. 6:**
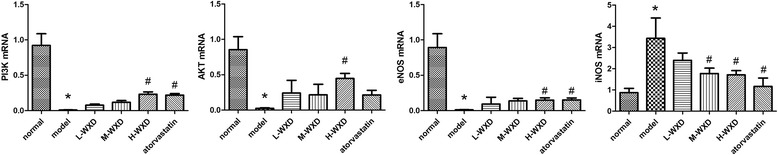
Fold-changes in the expression of PI3K, AKT, eNOS, and iNOS mRNA in the thoracic aorta, measured by real-time polymerase chain reaction (PCR) and calculated by the 2^−∆∆CT^ method, after treatment with various doses of Wen-Xin Decoction (WXD) for 30 days. Data are obtained from six rats. *p < 0.05 compared with the normal group. ^#^ *p* < 0.05 compared with the model group

Compared with those in the normal group, mRNA levels of PI3K, AKT, and eNOS were significantly decreased in the model group (Fig. 6), while compared with those in the model group, these levels were increased in the high-dose WXD group. The effects of WXD treatment were dose dependent. Compared to the model group, high-dose treatment increased PI3K, AKT, and eNOS mRNA expressions by 27.2 (*p* < 0.05), 18.8 (*p* < 0.05), and 14.5 folds (*p* < 0.05), respectively. Meanwhile, it downregulated iNOS mRNA expression by 0.50 folds compared with atorvastatin.

## Discussion

The NO level should be within the physiological range for effective endothelial protective effects; very high or very levels can cause vascular endothelial damage. Therefore, we analyzed NO levels and the NO/ET-1 ratio using quality control analysis methods in rat models of AS in the present study conducted to assess the protective effects of WXD on the vascular endothelium. We first obtained Mean ± σ data for the normal group and defined the normal range values as Mean ± 2σ. Then, we compared the values obtained for each group with the normal value to determine whether the data were within the normal range. We successfully created a rat model of AS by intraperitoneal injections of large doses of Vitamin D3 and the administration of a high-fat diet for 5 consecutive months. This combination resulted in intimal damage, deformation and loss of endothelial cells in the thoracic aorta, a significant decrease in the expression of serum NO levels, and a balanced NO/ET-1 ratio. WXD treatment significantly decreased the extent of vascular endothelial damage and promoted the recovery of endothelial cell morphology. These effects may be attributed to the increase in NO levels within the physiological range and a balance in the NO/ET-1 ratio. High-dose WXD exhibited better protective effects compared with atorvastatin.

We further demonstrated that the effects of WXD treatment on NO expression may be achieved in two ways. First was the promotion of NO expression within the physiological range through regulation of the PI3K/AKT pathway and activation of its downstream target eNOS gene expression. WXD significantly increased the expression of PI3K, AKT, and eNOS at both gene and protein levels. Meanwhile, it significantly promoted eNOS and AKT phosphorylation, which in turn activated the PI3K/AKT/eNOS pathway to promote NO expression within the physiological range. Second was a decrease in iNOS protein expression to inhibit the excessive generation of NO and ET-1. Thus, WXD simultaneously increased NO expression within the physiological range to relax blood vessels and prevent vascular endothelial damage caused by excessive NO production. Therefore, WXD modulates NO via eNOS and iNOS dual regulation.

NO synthesis and release is regulated by NOS, including eNOS and iNOS. A meta-analysis indicated that, in endothelial cells, functional eNOS oxidized its substrate L-arginine to L-citrulline and NO, which is the main source of NO in endothelial cells. This mechanism is known as the eNOS/NO synthesis pathway. When eNOS is activated, it produces NO that diffuses to vascular smooth muscles and increases intracellular cAMP concentration by activating soluble guanylate cyclase, thus playing a role in blood vessel dilation, inhibition of platelet and monocyte cell adhesion [15], and the maintenance of normal physiological functions of endothelial cells. Studies have shown that the eNOS activity can be regulated at both protein expression [16] and gene transcription levels, including eNOS phosphorylation and the interactions of other active substances [17, 18]. Activation of the PI3K pathway can enhance eNOS phosphorylation [19]. Studies have demonstrated that PI3K/AKT can promote eNOS/NO expression in the cardiovascular system and play a role in the protection of endothelial function; meanwhile, endothelial dysfunction is related to inhibition of the PI3K/AKT/eNOS pathway [20]. The results from this study further confirmed that WXD protects the vascular endothelium via regulation the PI3K/AKT/eNOS pathway.

The pathological process of AS, from the onset of disease to plaque formation and rupture, develops over the course of decades. This process involves extremely complex cell signal transduction pathways. A specific pathway may only be activated in a certain period during the pathogenesis cycle, and the same pathway activated at different time points may act like a “double-edged sword.” The PI3K/AKT pathway is one such pathway in AS pathogenesis. For example, activation of the PI3K/AKT pathway can inhibit vascular smooth muscle cell migration [21], decrease platelet adhesion [22, 23], inhibit tissue factor expression, decrease vascular endothelial cell apoptosis [24, 25], and promote eNOS/NO expression. On the other hand, activation of PI3K in macrophages via oxLDL and Ang II can increase macrophage activity and enhance the immune inflammatory response [26, 27]. In the present study, we demonstrated that WXD can activate the PI3K/AKT/eNOS pathway; however, the effects of WXD treatment on macrophage activity remain elusive and will be the focus of our future research.

## Conclusion

In conclusion, our results suggest that WXD protects and maintains the integrity of the vascular endothelium by activating the PI3K/AKT/eNOS pathway, decreasing iNOS expression, and promoting the release of physiological NO levels. Safe and effective treatment of CHD remains a worldwide problem. Classic thrombolytic therapy or modern bypass techniques fail to decrease the mortality from CHD. Chinese medicine has been clinically used for more than 1000 years and has been proven to be safe and effective. The effects of WXD on the treatment of CHD have been studied for nearly 30 years by Professor Cao Hongxin. In the present study, we further demonstrate that WXD increases the physiological NO levels to protect the vascular endothelium in AS, thus providing a possible alternative for the treatment of CHD.

## Abbreviations

AKT/PKB: protein kinase B
Ang II: angiotensin II
AS: atherosclerosis
CHO: cholesterol
eNOS: endothelial nitric oxide synthase
ELISA: enzyme-linked immunosorbentassay
ET-1: endothelin-1
HDL-C: high-density lipoprotein
iNOS: inducible nitric oxide synthase
LDL-C: low-density lipoprotein
NO: nitric oxide
ox-LDL: oxidized low density lipoprotein
Real-time PCR: real-time polymerase chain reaction
PI3K: phosphatidylinositol 3-kinase
TG: triglyceride
VSMC: vascular smooth muscle cell
